# Clinical Profile and Outcomes in Anti-TIF1γ Positive Idiopathic Inflammatory Myositis Patients: A Greek Cohort Study

**DOI:** 10.31138/mjr.300525.iao

**Published:** 2025-06-30

**Authors:** Vasiliki Syrmou, Christos Liaskos, Eleni Patrikious, Ioannis Alexiou, Theodora Simopoulou, Christina G. Katsiari, Dimitrios P. Bogdanos

**Affiliations:** Department of Rheumatology and Clinical Immunology, Faculty of Medicine, School of Health Sciences, University of Thessaly, University General Hospital of Larissa, Larissa, Greece

**Keywords:** paraneoplastic syndrome, malignancy, dermatomyositis, myositis-specific antibodies, cancer-associated myositis

## Abstract

**Background:**

Anti-transcription intermediary factor 1-gamma (anti-TIF1γ) antibodies are closely associated with Inflammatory myositis (IIM) and cancer-associated myositis.

**Objective:**

Description of clinical characteristics of anti-TIF1γ(+) IIM patients in a Greek population.

**Material & Methods:**

Retrospective analysis with 113 IIM cases between 2001 and 2024 was performed and clinical and laboratory data were collected. Disease manifestations and outcomes were compared between anti-TIF1γ-positive and -negative groups.

**Results:**

Twenty patients (17.7%) were anti-TIF1γ(+), of which 70% were women. The mean age was 64.8 ± 12.5 years vs 59.61 ± 12.81 of anti-TIF1γ(−) patients (p>0.05). Anti-TIF1γ was strongly associated with Dermatomyositis (DM) (95%, p < 0.001) and more severe cutaneous involvement (mean CDASI=27.35 ± 15.01 vs 14 ± 12.25 p =0.0015). Malignancy was significantly more frequent in the anti-TIF1γ(+) group (60% vs. 20.4%, p = 0.001), with an odds ratio of 5.84 (95% CI 2.09–16.31). Logistic regression identified anti-TIF1γ positivity as independent predictor of malignancy. Interstitial Lung Disease was uncommon among anti-TIF1γ(+) cases (15%, p = 0.004), while dysphagia was far more prevalent (55% vs. 22.6%, p = 0.001). Muscle power (MMT-8score) and CPK levels did not differ significantly, and survival was lower in anti-TIF1γ(+) patients (55.7% vs. 82.6% p<0.001), associated with malignancy.

**Conclusions:**

In our cohort, anti-TIF1γ antibodies define a distinct IIM subset marked by severe skin disease, high malignancy risk, and poorer survival, supporting comprehensive cancer screening and tailored immunosuppressive treatment. This study describes this phenotype in a Greek cohort, aligning with international evidence and highlighting the need for collaborative studies.

## INTRODUCTION

Idiopathic Inflammatory myopathies (IIM) are a heterogenous group of autoimmune conditions characterised mainly by muscle destruction and inflammation, commonly including extra muscular manifestations like typical skin rash, interstitial lung disease (ILD), dysphagia, and pulmonary hypertension. IIM are further classified depending on clinical and immunologic markers to dermatomyositis (DM), polymyositis (PM), inclusion bodies myositis (IBM), immune-mediated necrotising myopathy (IMNM), anti-synthetase syndrome (ARS) and overlap myositis.^1–8^ It is well described in literature that IIM is related to malignancy, with malignancy prevalence in IIM patients ranging between 6.8 and 32%, with some of the subtypes showing closer association.^9–13^ DM seems to carry the highest risk for underlying malignancy with a standardised incidence ratio for malignancy up to 5.5.^14,15^

Several autoantibodies have been described so far in patients with IIM, each of them associated to specific IIM subtype encouraging prompt diagnosis and identification of distinct disease patterns. Anti-TIF1γ carries the highest risk, with up to 84% of positive patients having underlying malignancy.^13,16–20^ TIF1γ, also known as ectodermin, is a protein with multiple roles involved in transcriptional regulation, posttranslational modifications, and protein degradation.^8,19,20^ Encoded by the TRIM33 gene on chromosome 1p13,^20^ TIF1-γ belongs to the TIF1 family alongside TIF1-a, TIF1-b, and TIF1-d^21^ and is involved in cell cycle regulation, β-catenin degradation, DNA repair,^22^ and immune response modulation by interfering in macrophage activation and inflammas- ome complex formation.^23,24^ Through E3 ubiquitin and SUMO ligase activities, it has a role in posttranslational level for several proteins.^25^ Its role in tumorigenesis has garnered significant research interest, particularly in haematological and epithelial cancers.

The present study aimed to describe in great detail the laboratory and, particularly, the clinical features and outcome of anti-TIF1γ positive patients in our Greek cohort of IIM.

## MATERIALS AND METHODS

Hospital records of all IIM patients treated in the Department of Rheumatology and Clinical Immunology, University General Hospital of Larissa, a tertiary and referral centre for rheumatic diseases in Central Greece between 2001 and 2024, were retrospectively reviewed. This study comprised only IIM patients classified based on the 2017 EULAR/ACR classification criteria.^3,5^ Data were collected on patient gender, myositis subtype (DM, PM, ARS, overlap myositis, IMNM), age at myositis diagnosis, age at malignancy diagnosis, and involvement of muscles, skin, lungs, and gastrointestinal system, as well as basic comorbidities. Our study protocol, consistent with the Helsinki Declaration, was approved by the Ethics Committee of the University General Hospital of Larissa (protocol No 48685/28/11/2024). More specifically, skin involvement was assumed to indicate the presence of a classic dermatomyositis rash. Evidence of Gottron’s papules, Gottron’s sign, heliotrope sign, V-sign, shawl sign, facial erythema, periorbital oedema/erythema, alopecia, depigmentation, scarring, telangiectasias, Mechanic’s hands, Hiker’s feet, photosensitivity, Holster sign, periungual telangiectasias, and mucosal involvement was recorded. Livedo reticularis and Raynaud’s syndrome were also documented but were not classified as skin involvement. The CDASI score was calculated for both activity and damage, based on the assessment of medical photography. Muscle involvement was recorded as positive when muscle biopsy, MRI, or electromyography (EMG) provided evidence of muscle inflammation. The MMT-8 score was calculated, and the creatine phosphokinase (CPK) level at diagnosis (mg/dL) was noted. Lung involvement was considered positive if imaging of the thorax (High-Resolution Computed Tomography) showed evidence of an inflammatory or fibrotic process. The percentage of predicted forced vital capacity (FVC) adjusted for BMI was recorded, along with the imaging pattern, specifically noting the presence of Non-Specific Interstitial Pneumonia (NSIP), Usual Interstitial Pneumonia (UIP), honeycombing, or emphysema. For gastrointestinal involvement, medical records were screened for evidence of swallowing difficulties, and changes in voice were logged. Data regarding comorbidities including hypertension (HTN), hyperlipidaemia, diabetes, coronary artery disease, stroke and peripheral arterial disease, atrial fibrillation, hypothyroidism, hyperthyroidism, chronic kidney disease (CKD), depression, aortic aneurysm, acute myocardial infarction, and other autoimmune diseases were reviewed. The autoantibody profile was assessed, including conventional indirect immunofluorescence for ANA using Hep-2 cells as substrate, ELISA for anti-ENA, and myositis profile line blot analysis (Euroimmun, Lübeck, Germany) testing MSA and MAA for a range of autoantibodies: anti-Mi2a, anti-Mi2b, anti-TIF-1γ, anti-MDA5, anti-NXP2, anti-SAE, anti-Ku, anti-PM-Scl100, anti-PM-Scl75, anti-Jo1, anti-SRP, anti-PL7, anti-PL12, anti-EJ, anti-OJ, anti-Ro52,, anti-CN1a, anti-HA, anti-Ks, and anti-Zo. Finally, levels of ferritin, haemoglobin (Hb), white blood cells (WBC), and lactate dehydrogenase (LDH) in serum were collected.

## STATISTICAL ANALYSIS

Statistical analysis was conducted for demographics, organ involvement, disease subtypes, and autoantibodies. All statistical calculations were performed using SPSS 25 program while graphs were designed using Graph Pad Prism Software. Differences between groups were tested by chi-square test and Fisher’s Exact Test (2-sided), two tailed t-test (for Equality of Means, equal variances are not assumed) and the non-parametric Mann-Whitney Test, as well as Kaplan Meier survival curves. Fisher’s exact test was used followed by post-hoc analysis for contingency tables. Logistic regression analysis was done for independent risk factors of malignancy. Venn diagram was created using InteractiVenn online tool. A p-value less than 0.05 was considered statistically significant.

## RESULTS

**Figure 1.**
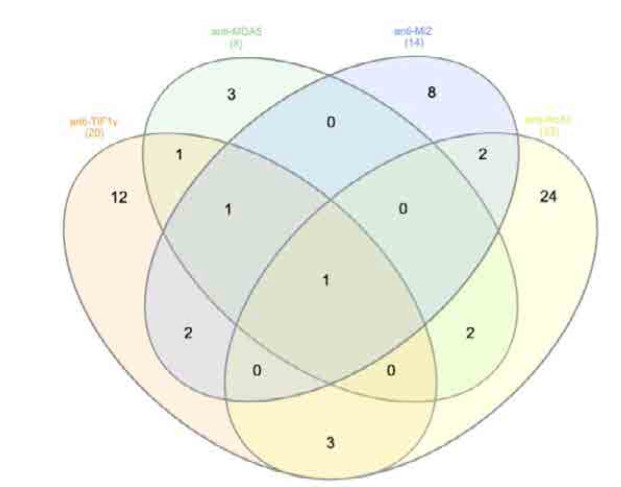
Venn diagram illustrating the coexistence of anti-TIFγ with anti-MDA5, anti-Mi2, anti-Ro52 antibodies.

**Figure 2.**
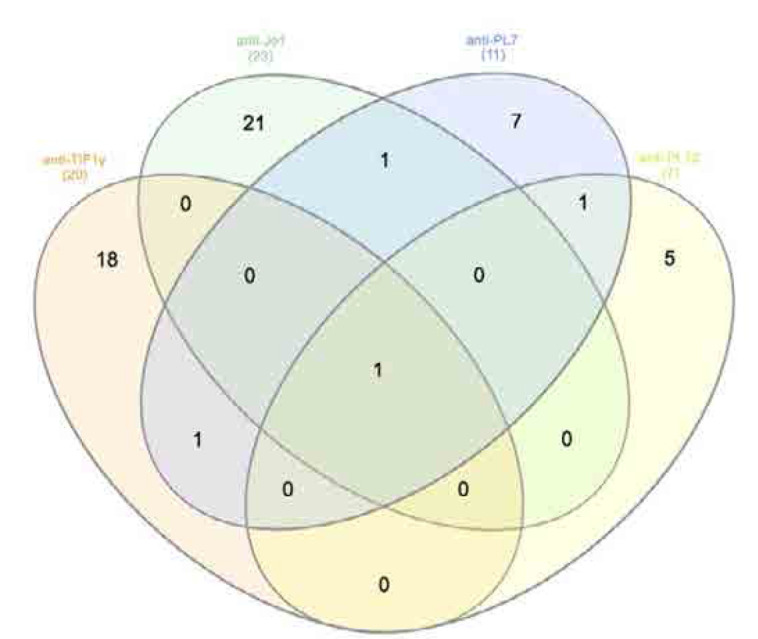
Venn diagram illustrating the coexistence of anti-TIF1γ with anti-Jo1, anti-PL7, anti-PL12.

We identified 113 cases of myositis, of which 20 (17.7%) patients were anti-TIF1γ positive, 18 (90%) of whom were also positive for ANA by indirect immunofluorescence. The mean levels of arbitrary units of anti-TIF1γ (+) cases were 41.1 ± 21.16 SD. In 3 cases, anti-MDA5 was concurrently detected, in 4 cases anti-Mi2, 1 had anti-Ku, 1 had anti-Pm-Scl 75, 1 had anti-Jo1, 2 had anti-PL-7, 1 had anti-PL-12, 4 cases had anti-Ro52, and 2 cases had anti-ENA. Co-occurrence of various autoantibodies with anti-TIF1γ is depicted as Venn diagrams (**Figure 1** and **Figure 2**).

### Age and Gender

Fourteen cases were women (70%), but there was no significant statistical correlation between gender and anti-TIF1γ (**Table 1**). In terms of age at diagnosis of myositis, in the anti-TIF1γ (+) cases the mean age was 64.8 years ± 12.49 vs 59.61 ± 12.81 SD of anti-TIF1γ(−) patients (p=ns). The main demographic, clinical and laboratory characteristics of anti- anti-TIF1γ(+) and anti-TIF1γ(−) patients are presented in **Table 1** and **Table 2**.

**Table 1.** Main demographic, laboratory and clinical characteristics of anti-TIF1γ (+) and anti-TIF1γ (−) patients.

**Clinical Characteristics**	**Anti-TIF1γ + (n=20)**	**Anti-TIF1γ (n=93)**	**p-value**
Female (%)	14(70)	61 (65.6)	0.105
Age (mean ± SD)	64.8 ± 12.49	59.61 ± 12.81	0.906
Skin Involvement	19/1	46/47	**<0.001^a^**
Type of disease (DM/PM/ARS/OM/IMNM)	19/1/0/0/0	28/10/35/14/6	<0.001^*^
Malignancy (yes/no)	12/8	19/74	**0.001**
Lung involvement	3/17	48/45	**0.004^a^**
Raynaud’s Phenomenon	3/17	28/65	0.269^a^
Periungual erythema	11/9	32/61	0.142^a^
Livedo Reticularis	2/18	18/75	0.519^a^
Mechanic’s Hands	5/15	14/79	0.454^a^
Muscle involvement	20/0	83/10	0.27^a^
CPK (mg/dl) (mean ± SD)	1350 ± 1452	2338 ± 3671	0.988^b^
MMT-8 (mean ± SD)	117.5 ± 22	127.7 ± 20.9	0.069
Dysphagia	11/9	21/72	**0.001**
Voice change	8/12	19/74	0.116
Arthritis/Arthralgias (yes/no)	10/10	59/34	0.387
LDH (mg/dl) mean ±SD	482.8 ± 245.2	474.2 ± 276.4	0.89
CRP (mg/dl) mean ± SD	1.6 ± 1.49	2.5 ± 3.39	0.067
ESR (mm/h) mean ± SD	43.8 ± 22.6	45 ± 59.5	0.873
Ferritin (mg/dl) mean ± SD	595.9 ± 667.3	580.1 ± 903.8	0.929
WBC	7775 ± 4777	8,824 ± 3,543	0.362
Hb (g/l)	12.24 ± 1.4	12.27 ± 1.57	0.914

DM: Dermatomyositis; PM: Polymyositis; ARS: Antisynthetase syndrome; OM: Overlap Myositis; IMNM: Immune Mediated Necrotizing Myositis; FVC: Forced Vital Capacity; NSIP: Non Specific Interstitial Pneumonia; UIP: Usual Interstitial Pneumonia; HC: Honeycombing; EMPH: Emphysema; CPK: Creatine Phosphokinase.

* Contingency table was performed followed by chi-square, Fisher’s exact test and post hoc analysis revealing maximum significance for DM especially when compared to ARS p<0.001, (DM vs OM p=0.053, DM vs IMNM p=0.78, DM vs PM p=1, PM vs ARS p=1, PM vs OM p=1, PM vs IMNM p=1, ARS vs OM p=1, ARS vs IMNM p=1, OM vs IMNM p=1)

** Contingency table was performed followed by chi-square, Fisher’s exact test and post hoc analysis revealing maximum significance for nil pattern (normal) especially when compared to NSIP p=0.046, (nil vs UIP p= 0.56, nil vs HC p=1, nil vs emph p=1, UIP vs NSIP p=1, NSIP vs HC p=1, NSIP vs emph p=1, UIP vs HC p=1, UIP vs emph p=1, HC vs emph p=1).

a p- Values were calculated using Fisher’s exact test (2-sided).

b p-Values were calculated using Mann-Whitney test.

**Table 2.** Detailed characteristics of skin signs among patients with skin involvement in anti-TIF1γ (+) and anti-TIF1γ (−) IIM patients.

**Main skin signs**	**Anti-TIF1γ(+) (n=19)**	**Anti-TIF1γ(−) (n=46)**	**p-value**
CDASI total score	27.45 ± 13.93	14 ± 12.25	**0.0015^b^**
Gottron’s sign (yes/no)	17/2	25/21	**0.009^a^**
Gottron’s papules	19/0	27/19	**<0.001^a^**
V-sign	16/3	34/12	0.52^a^
Shawl sign	11/8	17/29	0.17
Heliotrope sign	18/1	31/15	**0.02^a^**
Photosensitivity	16/3	28/18	0.08^a^
Skin Ulceration	8/11	11/35	0.22
Holster sign	7/12	6/40	**0.042^a^**
Facial Erythema	14/5	31/15	0.77
Alopecia	4/15	8/38	0.73^a^
Scarring	3/16	2/44	0.14^a^
Depigmentation	5/14	11/35	1^a^
Mechanic’s Hands	5/14	9/37	0.52^a^
Periorbital oedema	16/3	29/17	0.14^a^
Mucosal lesions	4/15	10/36	1^a^
Sleeve sign	4/15	10/36	1^a^

There were 65 patients with skin involvement (46 with DM and 11 with ARS and 8 with OM).

CDASI: Cutaneous Dermatomyositis Disease Area and Severity Index.

a p- Values were calculated using Fisher’s exact test (2-sided).

b p-Values were calculated using Mann-Whitney test.

### Malignancy

Twelve anti-TIF1γ(+) cases (60%) had malignancy compared to 19 cases of malignancy in the anti-TIF1γ(−) group (20.4%), (p=0.001) revealing a significant correlation between the autoantibody detection and the diagnosis of malignancy. The mean levels of anti-TIF1γ(+) cases with malignancy did not differ to those without malignancy (34.75AU ± 14.41SD vs 45.33AU ± 25.05SD). Assessing the risk for malignancy among the positive anti-TIF1γ cases it is nearly six times higher comparing to the negative cases (Odds ratio=5.84 with a 95% confidence interval (CI) of 2.09 to 16.31 p=0.0027). Among anti-TIF1γ(+) patients with malignancy (n=12), 4 cases had breast cancer, 2 lung cancer, 1 colorectal, 1 ovarian, 1 prostate, 1renal cell and 1 bladder urothelial carcinoma and 1 oropharyngeal carcinoma. In 5 cases, the diagnosis of malignancy was synchronous to myositis (41.7%), in 4 cases the myositis preceded (33.3%), and in 3 cases it followed the diagnosis of malignancy (25%). Six patients were diagnosed with an advanced stage of malignancy (stage III or IV), and 6 with early stages. Finally, all cases with malignancy originated from solid organs, the most common type being adenocarcinoma (n=10, 83.3%). Logistic regression analysis for malignancy risk revealed statistical significance for TIF-1γ status (OR=5.84 CI= 2.09 –16.31 p=0.0027) for age (OR=1.09 CI=1.04–1.14 p<0.001) setting those 2 parameters as independent risk factors for malignancy.

### Skin involvement

Nineteen (95%) of the anti-TIF1γ(+) patients presented evidence of skin involvement vs 46 patients (49.4%) in the anti-TIF1γ(−) group. Statistical comparison for difference in distribution of TIF1γ between myositis sub-types revealed significant difference (p<0.001) (**Table 1**). Post hoc-analysis further associated anti-TIF1γ autoantibody with DM (**Table 1**).

From the whole group, evidence of skin involvement was documented in 65 patients (46 with DM, 11 with ARS and 8 with OM). Analysis in those cases revealed significantly higher total CDASI score in anti-TIF1γ(+) vs anti-TIF1γ(−) patients (mean± SD: 27.45±13.93 vs 14±12.25, respectively, p=0.002) suggesting that anti-TIF1γ positivity is strongly associated with more severe skin disease at diagnosis. Analysis for specific skin signs showed significant correlation for Gottron’s papules, Gottron’s sign, heliotrope sign and Holster sign p<0.05 (**Table 2**).

Among patients with DM (n=46), 19 were TIF1γ (+) and 37 were TIF1γ (−). Total CDASI score was also found significantly higher in TIF1γ patients when compared to TIF1γ(−) patients (mean± SD: 27.45±13.93 vs 14±12.25, p=0.045). In terms of the typical skin signs, only Gottron’s papules are maintaining statistically correlation with this autoantibody (p=0.031).

### Muscle involvement

Frequency and severity of muscle involvement was not significantly different between anti-TIF1γ (+) and TIF1γ(−) patients. All cases in the anti- TIF1γ (+) group had muscle involvement (100%) compared to 89.2% anti-TIF1γ(−) patients. Mean MMT-8 score in the anti-TIF1γ(+) group was 117.5 ± 22.05mg/dl compared to 127.72 ± 20.92mg/dl in the anti-TIF1γ (−) group (p=0.069). Although MMT-8 scores may suggest that anti-TIF1γ(+) patients tend to have moderately lower muscle strength, CPK levels at myositis diagnosis were lower in anti-TIF 1γ(+) patients (1,350 mg/dl ± 1,452SD) compared to the anti-TIF1γ(−) group (2,338 mg/dl ± 3,671 SD), p= 0.288). Finally, the existence of anti-TIF1γ was not associated with arthritis or arthralgias (**Table 1**).

### Lung Involvement

Pulmonary manifestations were not common in the anti-TIF1γ(+) group. There were 3 cases (15%) of lung parenchymal changes on CT thorax in the anti-TIF1γ(+) group compared to 48 cases (52%) in the anti-TIF1γ(−) group (p=0.004). Anti-TIF1γ(+) cases had lower odds of lung involvement compared to TIF1γ negative cases (Odds ratio= 0.22 95% CI=0.06–0.78). Lung involvement in the anti-TIF1γ(+) group encompassed 1 case with NSIP, 1 with honeycombing and 1 with profound emphysema and no cases with UIP compared to 32, 2, 1 and 13 cases in the anti-TIF1γ(−) group, respectively (p<0.004). Regarding FVC% at myositis diagnosis there was no statistically significant difference between the two groups (86.5 ± 15.3 vs 83.9 ± 18.4, p= 0.506). CT thorax in the anti-TIF1γ(+) subgroup was normal in 85% compared to 48.4 % of cases in the anti-TIF1γ(−) group (Table 1). In our group of anti-TIF1γ(+) patients without evidence of lung involvement upon diagnosis, no new ILD manifestations were observed during their follow-up.

### Dysphagia and voice change

Difficulty in swallowing is very common (n=11, 55%) among patients with anti-TIF1γ autoantibody compared to patients without (n=21,22.6%, p=0.001). The experience of voice change did not differ amongst the two groups (**Table 1**).

### Comorbidities

No statistical correlation was identified between the existence of anti-TIF1γ antibody and any of the main comorbidities observed in our cohort (HTN, hyperlipidaemia, T2DM, CAD, stroke, AF, PAD, hyperthyroidism, hypothyroidism, CKD, depression, other autoimmune diseases, Aortic Aneurysm, Acute Myocardial Infarct, smoking, osteoporosis) (**Table 3**).

**Table 3.** Coexistence of comorbidities.

**Comorbidity**	**Anti-TIF-1γ (+) (n=20)**	**Anti-TIF1γ (−) (n=93)**	**p-value**
HTN(yes/no)	7/13	48/45	0.27
Hyperlipidaemia	4/16	37/56	0.126 **^a^**
T2DM	5/15	14/79	0.454
CAD	2/18	14/79	0.733 **^a^**
Stroke	0/20	3/90	1 **^a^**
PAD	0/20	11/82	0.208 **^a^**
AF	0/20	9/84	0.357 **^a^**
Hypothyroidism	3/17	23/70	0.558 **^a^**
Hyperthyroidism	1/19	2/91	0.446 **^a^**
CKD	1/19	13/80	0.458 **^a^**
Depression	2/18	18/75	0.519 **^a^**
Autoimmune diseases	2/12	13/80	1 **^a^**
Aortic Aneurysm	0/20	5/88	0.584 **^a^**
AMI	1/19	8/85	1 **^a^**
Smoking	4/16	30/63	0.421 **^a^**
Osteoporosis	8/12	47/46	0.543

HTN: hypertension; T2DM: Type 2 Diabetes Mellitus; CAD: Coronary Artery Disease; PAD: peripheral arterial disease; AF: Atrial Fibrillation; CKD: Chronic Kidney Disease; AMI: Acute Myocardial Infarct.

a p- Values were calculated using Fisher’s exact test (2-sided).

### LDH, ferritin, CRP, ESR, WBC, Hb levels

CRP levels presented to have a tendency towards lower values in cases with anti-TIF1γ(+) antibody compared to negative cases (1.6 ± 1.49 vs 2.5 ± 3.39, p=0.069). The other blood test values (LDH, ESR, Ferritin, WBC, Hb) did not show a significant correlation with TIF1γ status (**Table 1**).

### Survival

**Figure 3.**
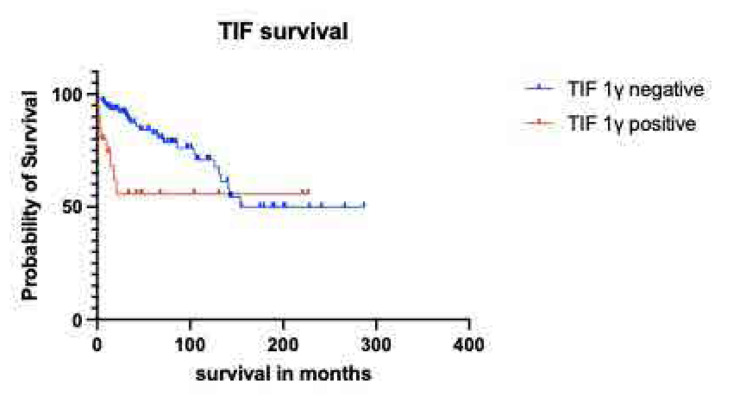
Kaplan-Meier survival curves comparing probability of survival in anti-TIF1γ (+) cases (red line) to anti-TIF1γ (−) cases (blue line) over time in months.

**Figure 4.**
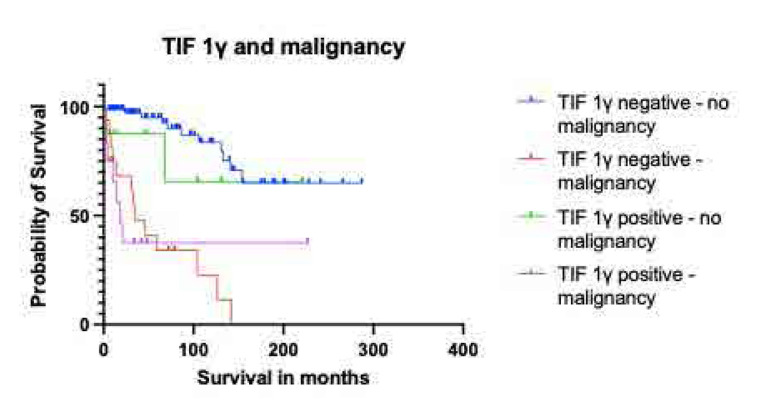
Kaplan-Meier survival curves comparing probability of survival in anti-TIF1 γ (+) cases with (purple line) or without (red line) malignancy and anti-TIF1γ (−) with (blue line) malignancy, vs anti-TIF1γ (−) without malignancy vs anti-TIF1γ (+) without (green line) malignancy over time in months.

The 6-month survival rate was 80% in the anti-TIF1γ (+) while in the anti-TIF1γ(−) group it was 96.8%, the 12-month was 74.3%. vs 94.5%, the 36 month was 55.7% vs 87.6%. In 5 years, the survival rate was 55.7% remaining stable in the positive group vs 82.6% in the negative group (p<0.001). Kaplan Meier curves are given in **Figure 3** and **Figure 4**. The leading cause of death was malignancy and only the anti-TIF1γ(+) cases that had no malignancy survived 5 or more years. Further analysis for malignancy revealed that median survival for anti-TIF1γ(+) cases with malignancy was 18 months.

**Figure 5.**
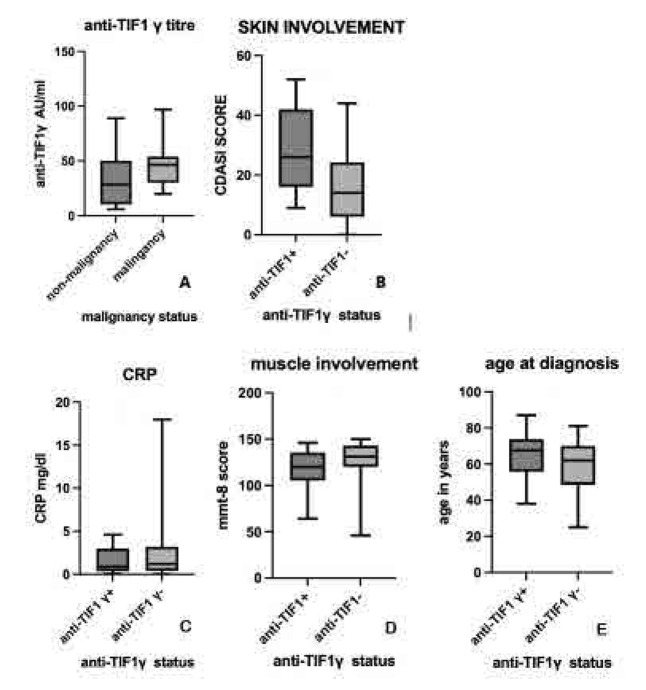
**A.** Box-plot diagram with mean + SD values of anti-TIF1γ titre in malignancy and non-malignancy cases in arbitrary units/ml (34.758AU/ml ±14.41SD vs 45.33 AU/ml± 25.05SD, p=ns). **B.** Box-plot diagram with mean CDASI total score, (mean 27.35±15.01SD vs mean 8.33 ±11.68SD, t-test 5.33, p<0.001). **C.** CRP in mg/dl (1.6±1.49 vs2.5±3.39, p=0.067). **D.** mean MMT-8 score score (mean= 117.5±22 vs 127.7±20.9, p=0.069). **E.** Age at diagnosis in anti-TIF1γ (+) vs anti-TIF1γ(−) cases (mean=64.8±12.49 vs 59.61±12.81, p=0.906).

### Treatment

Patients were treated conventionally and in accord with the established recommendations and clinical practise with various immunosuppressive treatment (**Table 4**) depending on malignancy status and the severity of clinical manifestations. In cases that malignancy preceded minimal treatment interventions were done like hydroxychloroquine 200mg. However, for severe cases with significant muscle weakness, IVIG in lower dose regime was given to have the minimal inhibitory effect on chemotherapy. In cases with severe manifestations (severe muscle weakness MMT8<110, dysphagia) with no obvious space occupying lesions on initial CT scan, treatment was more aggressive with Iv methylprednisolone pulses, IVIG and even cyclophosphamide as provided in **Table 4**.

**Table 4.** Immunosuppressive treatment administered in anti-TIF1γ (+) cases.

**Pt ID**	**Induction**	**Maintenance**	**2^nd^ Line**
pt1	1000mg MPD	MTX 10 mg	–
pt2	1500mg MPD, 100gr IVIG MTX 10mg	MPD 4mg MTX 10 mg +HCQ	GC+ AZA 150mg/MMF 3 gr
pt3	500mg MPD MTX 10 mg	MPD 4mg MTX 10 mg +HCQ	–
pt4	64 mg MPD MTX 15	MPD 4 mg MTX 10 mg	–
pt5	5000mg MPD 150gr IVIG	IVIG 50 gr/month for 5 months MTX 15 mg + HCQ	Stopped due to breast Ca
pt6	20 mg PRED	10 mg PRED	–
pt7	1500mg MPD 150 gr IVIG HCQ 200mg	30 mg PRED HCQ 200mg IVIG (600gr in total)	–
pt8	40 mg MPD + AZA 100 mg	AZA 100mg	MTX 15 mg
pt9	20 mg MPD HCQ 200mg	HCQ 200mg	–
pt10	2000mg MPD 160 gr IV Cyclosporin Cyclophosphamide 4000mg +150gr IVIG	MTX	AZA
pt11	60 mg MPD+ 60 gr IVIG+ HCQ 200mg	IVIG 120 gr HCQ	–
pt12	32 mg MPD +MTX 20 gr	MTX 15 mg	–
pt13	3000mg MPD+ 60 gr IVIG+ MTX 15 mg + HCQ 200 mg	MTX 15 mg +HCQ 200 mg	HCQ 200mg
pt14	3000mg MPD+ 125 gr IVIG+ Cyclophosphamide 500mg	–	–
pt15	3000mg MPD+ 100gr IVIG+ Cyclophosphamide 3000mg	MTX 15 mg IVIG 60 gr	AZA 100 mg
pt16	HCQ 200mg	HCQ 200mg	–
pt17	IVIG 150 mg	–	–
pt18	32 mg MPD+ MTX 15 mg+ HCQ 200mg	MTX 15 mg+ HCQ 200mg	–
pt19	3000mg MPD+ 60 gr IVIG+ Cyclophosphamide 5500mg	AZA 100mg	–
pt20	48 mg MPD+ 20gr IVIG+ AZA 100mg	AZA 100mg	–

Pt: patient; MPD: methylprednisolone; IVIG: human immunoglobulin; MTX: methotrexate; HCQ: Hydroxychloroquine; PRED: prednisolone; AZA: azathioprine; GC: glucocorticoids.

## DISCUSSION

To our knowledge, this is the first study describing in great detail the clinical profile and outcomes in anti-TIF1γ(+) IIM cases in a single-centre Greek cohort population. Our results revealed that the prevalence of anti-TIF1γ autoantibody among Greek IIM cases was 17.7%, while the detection of the autoantibodies was an independent risk for CAM along with age. Despite the small size of the group the Odds Ratio for CAM was 5.8, which agrees with the metanalysis results published by Best et al.^26^ In most cases, the diagnoses of the two entities were synchronous (within 3 months) while the rest of the cases were evenly distributed in the 5-year interval prior and following IIM diagnosis. This aligns with the findings presented in existing literature.^27,28^ Notably, all malignancies identified in our anti-TIF1-γ(+) cases were solid organ tumours, further corroborating published findings.^26^ Breast cancer was the most observed malignancy as seen in other groups.^29–31^ As observed in Greek population based on national registry for malignancies, breast cancer is the most common malignancy in women (33%) which probably explains the preponderance of this type in anti-TIF1γ(+) malignancy cases.^32^ This finding was observed also in other myositis national registries.^29^ In terms of ovarian cancer, which has been found to be disproportionally more common in the UKMyoNet no such conclusion can be made in our group where only one patient was identified probably due to sample size. Finally, regarding oropharyngeal and nasopharyngeal carcinomas there is evidence from Asian groups that is linked to CAM anti-TIF1γ(+).^33^ In our group, there was only one case and therefore conclusions are not safe. However, this malignancy type is not as common in Greece as in Taiwan.^29,31,33,34^

DM was the prevailing IIM subtype amongst anti-TIF1γ(+) cases (95%). The findings of our study align with the existing literature, reinforcing its striking significance despite the small size of the current series.^12,18,28, 35–41^ Moreover, in our group, anti-TIF1γ(+) patients appeared to have significantly more active skin disease, as seen by the markedly higher CDASI score comparing to negative cases not only among patients with skin involvement (DM plus some ARS plus some OM cases) but also in the pure DM subgroup itself. The hallmarks of skin DM signs are disproportionally encountered in the positive patients confirming the concurrence of the autoantibody with DM phenotype. On the contrary, ILD was not a common manifestation; dysphagia was far more commonly observed in the TIF-1γ group. Muscle involvement was common, however, moderately severe as implied by MMT-8 score and CPK levels. These clinical features are congruent with other cohorts and seem to comprise the autoantibody-associated- syndrome.^42^ To this date, there is only one study referring to Greek population with IIM cases that includes 7 cases of anti-TIF1γ patients. This study, although not focused on the anti-TIF1γ and being rather cross sectional included 1 case with DM, 3 with amyopathic DM and 3 with PM.^43^ In our group, all patients exhibited muscle involvement. According to the literature, the majority of anti-TIF1γ (+) patients develop muscle involvement, but patients with amyopathic have also been identified.^44^

The striking association of this autoantibody with malignancy has prompted investigators to suggest proper and thorough malignancy screens in the anti-TIF1γ (+) patients. This is reflected in the IMACS 2023 and other consensus statements.^27,28,37,45^ In terms of treatment, the basic approach depends on malignancy risk assessment and severity of manifestations. The presence of profound muscle weakness and severe dysphagia were the two key findings in our cohort of patients that encouraged decisions towards more aggressive immunosuppressive management. In this case, high dose corticosteroids have been used in iv. pulses. IVIG can be exceptionally valuable in cases with severe manifestations, where underlying malignancy is highly possible or confirmed as it does not seem to interfere with chemotherapy. Conventional DMARDS and alkylating agents have been also used in the absence of malignancy. In cases with confirmed malignancy, hydroxychloroquine, was an option for skin damage amelioration, with or without corticosteroids. For refractory cases IVIG – even in a smaller dose – has been used.^10,28,36,46^

There is circumstantial but also contradictory evidence that TIF1γ can either promote or inhibit malignancy. Some studies support a rather protective role of TIF1γ against malignancy via mono-ubiquitination of SMAD4 and subsequent inhibition of TGFβ-induced epithelial-to-mesenchymal transition.^20^ However, its deactivation via mutations, epigenetic modifications, or non-coding RNAs can encourage various tumours growth.^47–49^ Conversely, TIF1γ overexpression, particularly in SMAD4-deficient cells, has been linked to tumour promotion by inhibiting TGF-β-mediated apoptosis.^20^ Additionally, TIF1γ interferes with Wnt/β-catenin pathway as it promotes β-catenin degradation affecting gene transcription,^23^ while its overexpression has been linked to mitotic defects and chromosomal instability leading to more sinister tumour behaviour^50^ and highlighting its complex role in oncogenesis. The significant presence of anti-TIF1γ autoantibodies in cancerous patients implies that either an antigen-drive autoreactive response due to cryptic epitopes or a broader breakdown of immunological tolerance, which needs further investigation.

In conclusion, this is the first focused study of the clinical characteristics of anti-TIF1γ antibody positive patients with IIM in Greek population, largely aligning with the published literature from other parts of the world. These common characteristics suggest that multi-centre, national and international collaborations could pave the way for meaningful research in this rare disease.

## CONFLICT OF INTEREST

The authors declare no conflict of interest relevant to this study.

## FUNDING INFORMATION

None.
